# Unresectable Hepatic Metastasis of Uveal Melanoma: Hepatic Chemosaturation with High-Dose Melphalan—Long-Term Overall Survival Negatively Correlates with Tumor Burden

**DOI:** 10.1155/2020/5672048

**Published:** 2020-09-02

**Authors:** R. Brüning, M. Tiede, M. Schneider, P. Wohlmuth, H. Weilert, K. Oldhafer, A. Stang

**Affiliations:** ^1^Radiology and Neuroradiology, Asklepios Hospital Barmbek, Ruebenkamp 220, 22307 Hamburg, Germany; ^2^Biostatistics, ProResearch, Asklepios Hospital St. Georg, Lohmuehlenstrasse 5, 20099 Hamburg, Germany; ^3^Oncology, Asklepios Hospital Barmbek, Ruebenkamp 220, 22307 Hamburg, Germany; ^4^Faculty of Medicine, Semmelweis University Campus Hamburg, Lohmuehlenstrasse 5, 20099 Hamburg, Germany; ^5^Surgery, Asklepios Hospital Barmbek, Ruebenkamp 220, 22307 Hamburg, Germany

## Abstract

**Introduction:**

Percutaneous hepatic perfusion with melphalan (PHP-M) for hepatic metastasis of uveal melanoma (LMUM) achieves high local response rates, but the individual clinical benefit is poorly defined. We aimed to determine cofactors of response and clinical outcomes including the probability of long-term (5-years) overall survival (OS) in PHP-M-treated patients with LMUM. *Patients and Methods*. We retrospectively reviewed clinicopathological, radiological, and outcome data of 19 patients with unresectable LMUM treated with 43 PHP-M (median 2 PHP-M) between 2014 and 2019. Tumor response and adverse events were evaluated using RECIST 1.1 and the Clavien–Dindo classification. Kaplan–Meier methods and Cox regression hazard proportional models were used.

**Results:**

Of 19 patients, 10 (53%) achieved a partial response (PR) and 9 (47%) had stable disease (SD). There was no progressive disease (PD) and no adverse events exceeding Clavien–Dindo grade IV. Median OS was 16.7 months after the first PHP-M treatment and 26.4 months after initial diagnosis. Low hepatic tumor volume (median of 10 mL vs. 150 mL) was an independent predictor of favorable OS (hazard ratio (95% confidence interval): 0.190 (0.041, 0.893); *p* < 0.05), and female patients were at a lower risk compared with males (0.146 (0.017, 1.240)). Estimates of the overall survival were 0.213 (0.0449, 1) from first imaging (95% confidence interval) to 5 years and 0.793 (0.609, 1) and 0.604 (0.380, 0.960) for 1 and 2 years after chemosaturation, respectively. *Discussion*. PHP-M for nonresectable LMUV provides a safe and locally efficient liver-directed procedure that offers patients a chance for long-term OS, especially for patients with a low hepatic tumor burden.

## 1. Introduction

Metastatic uveal melanoma (UM) carries a poor prognosis with 1-year survival rates reported as low as 10–25% [1, 2]. Up to 50% of uveal melanomas develop hepatic metastasis [3] with reported median survival ranging from 4 to 15 months [4], suggesting a role for liver-directed local therapy in patients without extrahepatic disease.

No effective adjuvant systemic therapy has been demonstrated to reduce the risk of metastasis, as stated by Triozzi and Singh [5]. A meta-analysis of 29 phase II trials in metastatic uveal melanoma until 2015 aimed to define progression-free survival (PFS) and OS. Poor outcomes such as a median PFS of 3.3 months (6-month PFS 27%) and median OS of 10.2 months (1-year OS 43%) were described across all treatment groups based on more than 900 patients [6].

Immune checkpoint inhibitors, such as ipilimumab, nivolumab, and pembrolizumab, achieve long-term OS with 5-year OS rates of 34–52% in metastatic cutaneous melanoma [7–9] but yield poor outcome results in metastatic uveal melanoma (UM) with a median OS of 10–14 months [10–13]. At present, there is no standard therapy for metastatic UM, and the limited activity of systemic therapy compared with cutaneous melanoma poses a treatment challenge.

Percutaneous isolated hepatic perfusion with melphalan (PHP-M) has been licensed in the European Union (EU) with a CE mark. Although evidence to date is limited, liver-targeted therapies such as PHP-M chemosaturation hold promising results for patients in this setting. However, the OS reported so far was within a large range from 245 days to 27 months [14, 15], and data from randomized trials regarding long-term outcomes are not available.

To enable better stratification of patient selection for this procedure in the future, we sought to evaluate cofactors of prognosis in this setting using a retrospective search from our database. We also aimed to determine cofactors of response and clinical outcomes including the probability of long-term (5-years) OS in PHP-M-treated patients with LMUM.

## 2. Material and Methods

Patients: from 2014 to 2019, patients were treated by percutaneous PHP-M as a last-line therapy in our center. All treatments were recommended by the multidisciplinary tumor board for clinical indication, and all cases were treated according to the Declaration of Helsinki of 1964. Informed consent was obtained. Patients' baseline and outcome data are given in Table 1.

For this evaluation, data were collected and evaluated in a retrospective fashion based on the medical records and the PACS database; these data are available on request.

Only patients with an Eastern Cooperative Oncology Group (ECOG) performance status of 0–1 and with adequate hematologic, renal, and hepatic function data (e.g. hemoglobin > 8 g/dL; platelets > 150 thsd/*μ*L, bilirubin ≤ 3 × upper limit of normal (ULN)) were included.

Distant extrahepatic metastasis exceeding 10 mm in lymph nodes or in relevant other locations, recent history of transient ischemic attacks, heart failure (left ventricular ejection fraction <40%), contraindications to general anesthesia, or significant chronic obstructive or restrictive pulmonary disorders were considered as contraindications for PHP-M.

The patients had to have at least one follow-up including an MRI- or CT-based restaging; patients lost to follow-up were not included in this evaluation (*n* = 1).

Seven patients received previous systemic treatment (most often pembrolizumab), four patients received transarterial chemoembolization (TACE) or transarterial chemoperfusion (not chemosaturation), and three patients received previous surgical of ablative therapy, and these therapies were terminated for either side effects or progressive disease. Six patients had no specified previous therapy.

### 2.1. Procedure

For the procedure itself, a dedicated filter system (CHEMOSAT® Second Generation; Delcath Systems Inc., New York, NY, USA) was used as previously described [16]. For arterial infusion, a 6 French catheter was inserted through the femoral artery into the common hepatic artery, and a 2.7 French microcatheter (ProGreat®, Terumo, Japan) was used for selective chemoperfusion of the liver. This transarterial chemoperfusion was performed with a dosage of 3.0 mg/kg ideal body weight up to a maximum dose of 220 mg of melphalan (Alkeran®). For venous access, a 18 French double-balloon catheter, inserted through the contralateral femoral vein, facilitated isolation of the hepatic inferior cava segment, the upper balloon by being inflated in the right atrium and being pulled back to cover the diaphragm portion of the inferior vena cava and the lower balloon being inflated in the hepatic portion of the vena cava below entries to the hepatic vein. A separate access in the right internal jugular vein was used for blood return. During the infusion phase, 500 cc of melphalan solution was administered at a rate of 0.4 mL/sec, with intermediate angiograms to ensure correct flow. As suggested by the manufacturer, the subsequent washout was performed with the filtration circuit running for 30 min after cessation of the arterial infusion. In order to maintain an activated clotting time (ACT) above 500 s, which was mandatory for safe extracorporeal hemofiltration, heparin was administered as needed.

These procedures were performed under general anesthesia. All patients were then supervised for the first night under intensive care, in order to monitor blood pressure, coagulation, access sites, and laboratory findings. On the day after the procedure, in our institution, G-CSF (Neulasta®) was given routinely.

### 2.2. Treatment Outcome

The treatment outcome was measured retrospectively according to the response evaluation criteria in solid tumors (RECIST 1.1) [17], based on the sum of diameter. The sum of diameter was tested as an independent predictor of OS. Therefore, either computed tomography (CT) or magnetic resonance imaging (MRI) was routinely performed at least every 3 months after PHP-M.

Overall survival was calculated from initial diagnosis and from the first PHP-M until the last available follow-up or death. Progression-free survival (PFS) was calculated and is given either from first imaging or from the first PHP. For tumor volume analysis, the patient cohort was stratified to approximately 10 mL tumor volume versus approximately 150 mL.

We monitored for adverse events and they were classified according to the Clavien–Dindo classification [18].

Tumor volume was determined using Advantage Workstation 4.1.2 (GE Healthcare). For volumetric evaluation, the venous phase was chosen, and the lesions were outlined manually. Volumetry was performed on CTs directly prior to chemosaturation. Portal branches and bile ducts were excluded by thresholding the outlined volume.

Tumor stability was defined as stable disease (SD according to RECIST 1.1) in comparison to partial response (PD according to RECIST 1.1).

### 2.3. Statistics

Continuous data were summarized as means ± standard deviations or as medians [25th and 75th percentiles] as appropriate. Categorical data were presented as *N* (%).

Overall survival was estimated with the Kaplan–Meier method. Univariable Cox proportional hazards models were used to associate the covariables to overall survival. Effects on mortality were presented with hazard ratios and 95% confidence intervals.

Univariable logistic regression models were used to relate age, gender, and tumor size to tumor stability (which is considered as an undesired event compared to partial remission). The logistic regression models were presented with parameter estimates, standard errors, odds ratios and 95% confidence intervals.

*p* values of likelihood ratio tests were presented. All *p* values were two-sided and a *p* value <0.05 was considered significant. All calculations were performed with the statistical analysis software R (R Core Team, 2018).

## 3. Results

In 19 patients, there were 43 procedures of chemosaturation with a median of 2 chemosaturation per patient. The interval between first and second PHP was on average of 119 days (SD of 145 days).

### 3.1. Overall Survival

**Figure 1:**
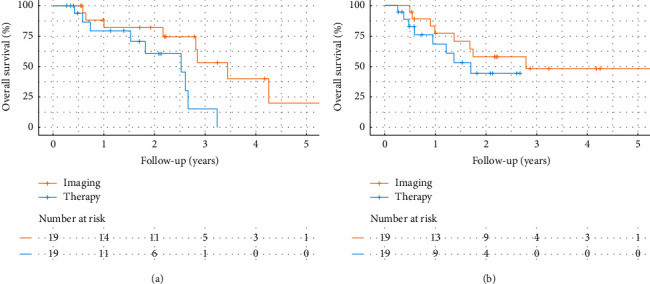
Kaplan–Meier plots. (a) Overall survival estimates (OS) (and patients at risk) from initial diagnosis/imaging and from initial chemosaturation therapy. (b) Estimates of progression-free survival (PFS) (and patients at risk) from initial diagnosis/imaging and from initial chemosaturation therapy.

Median OS following initial diagnosis was 26.4 months and following first PHP-M treatment was 16.7 month, as determined by the Kaplan–Meier method (Figure 1(a)). Progression-free survival (PFS) was 751.8 days since first imaging (SD 515.5 days) and 427.8 days since first PHP (SD 295.2 days) (Figure 1(b)).

**Figure 2:**
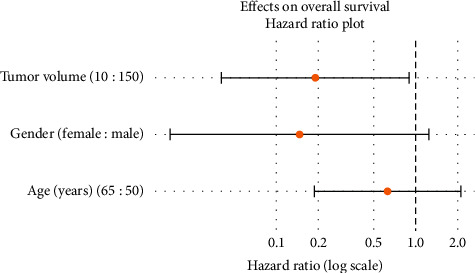
Plot of hazard ratios (95% confidence intervals) for overall survival.

**Figure 3:**
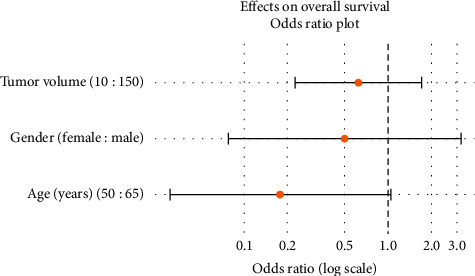
Plot of the odds ratios (95% confidence intervals) regarding tumor stability (tumor stability refers to stable disease RECIST 1.1, and compared to partial remission, it is referred to as an undesired event).

A lower tumor volume decreases the risk of mortality: increased OS was found associated with lower tumor volume (hazard ratio (95% confidence interval) for the tumor volume as stratified in 10 mL versus 150 mL: 0.190 (0.041, 0.893)). However, there was only a weak correlation between age and survival (HR (95% CI) for 65 versus 50 years: 0.627 (0.187, 2.100); Figure 2). The influence of gender was also estimated: in our patient cohort, the risk to females was only 15% of the risk to male patients (HR: 0.146 [0.017, 1.240]) (Figure 3). A correlation of OS to “time from initial diagnosis” showed a hazard ratio of 1.071 (lower/upper HR: 0.759/1.512), and the *p* value was not significant (0.695).

Elevation of serum LDH (in steps of 200 units) resulted in a hazard ration of 1.934 (lower/upper HR: 0.873/4.282); however, this trend was not significant (*p*=0.104). An increase in the sum of tumor diameter had a hazard ration of 2.503 (lower/upper HR: 0.732 8.556) in our cohort; but did not reach statistical significance (*p* 0.143).

Estimates of OS were 0.213 (0.0449, 1) from first imaging (95% confidence interval) to 5 years and 0.793 (0.609, 1) and 0.604 (0.380, 0.960) for 1 and 2 years after chemosaturation, respectively.

### 3.2. Restaging by RECIST

Ten of 19 patients achieved a partial response (PR; 53%), and 9/19 had stable disease (SD; 47%) following the initial treatment. There was no progressive disease (PD) and no complete response (CR).

For the statistical analysis, SD and tumor stability (compared to partial remission) were referred to as an undesired event. Tumor stability (and not partial response) was inversely correlated to lower age (odds ratio (95% confidence interval) of age = 50 years versus age = 65 years: 0.179 (0.031, 1.040)) (Figure 3). However, there was only weak evidence that female gender (OR (95% CI): 0.500 (0.078, 3.210)) or a lower tumor volume were associated with a marginally lower risk of SD (OR (95% CI): 0.623 (0.226, 1.710)).

### 3.3. Adverse Events

Adverse events according to the Clavien–Dindo classification until discharge: In our cohort of the 43 procedures, there were no or minor complications—grade ≤1 in 39 procedures, one case with grade II, in 2 cases grade IIIa (coronary ischemia), and in one case grade IIIb (transfemoral bleeding with following surgery); no grade IV-V occurred. Details are given in Table 2.

There were changes in the hematological state, most profoundly in platelet count (from 251.7/nL (SD 65.8) before the first PHP-M procedure to an average of 104.2/nL (SD 45.4) following the procedure). Bilirubin following the procedure was stable at an average of 0.9 (SD 0.6), and erythrocytes following the procedure were stable at 4.1/pL (SD 0.5).

## 4. Discussion

Uveal melanoma (UM) once metastatic carries a poor prognosis. Unfortunately, even modern agents such as immune checkpoint inhibitors—ipilimumab, nivolumab and pembrolizumab—yield poor outcome results in metastatic uveal melanoma (UM) with a median OS of 10–14 months [10–12]. At present, there is no standard therapy for metastatic UM, and the limited activity of systemic therapy compared with cutaneous melanoma poses a treatment challenge.

Of the metastatic UM, more than every second uveal melanoma develops hepatic metastasis [3], suggesting a role for liver-directed local therapy in patients without extrahepatic disease. As a consequence of this known predilection of uveal melanoma for hepatic metastasis, liver-directed therapies represent an important research focus in the treatment of metastatic disease [19, 20].

In our study treating unresectable liver metastasis with PHP-M and in part refractory to systemic therapy, median OS was 26.4 months following initial diagnosis and 16.7 months from first PHP-M treatment. Thus, we were able to show that PHP offers as second-line option the chance of the long-term OS outcome.

Our data on OS were compared positively with published reports. Karydis et al. reported an overall survival of 15.3 months following treatment in a larger retrospective bicenter evaluation with over 50 patients [21]. Data from a multicenter trial report a median OS of 9.6 months with a range from 1.6 to 41.0 months [19]. However, this work was a retrospective analysis of data acquired from multiple centers throughout Germany, resulting in a substantially higher heterogeneity of data [19]. An initial phase III trial of PHP with melphalan compared with best alternative care in 93 patients with ocular (88%) or cutaneous (12%) melanoma demonstrated an overall response rate with PHP-M therapy; OS was similar between the two arms; however, this was confounded by allowing crossover [22]. Further analysis on this patient cohort exhibited that while the hPFS was significantly better for the PHP-M group (*p* < 0.0001), the median OS was not significantly different (PHP-Mel 10.6 months vs. BAC 10.0 months) [23].

In our cohort, the main factor found to be beneficial for prolonged OS was lower tumor volume. This observation needs to be verified; however, no such tumor volume-associated analysis has, to the best our knowledge, been published. Also, the influence of gender was estimated: in our patient cohort, females had only 15% of the risk of male patients; this is in accordance with a recently published multivariate analysis on an international patient population that showed male patients (and those with elevated LDH and elevated ALP) were all associated with a shorter PFS [6]. In our patient cohort, there was only weak evidence that age was associated with survival. However, we did observe a trend that elevated serum LDH had an elevated hazard ration, but this did not reach statistical significance. Estimates of 5-year OS (95% confidence interval) from first imaging were 0.213 (0.0449, 1). This again may prove a strong argument toward the use of PHP-M.

In this retrospective single-institutional study, we observed a disease control in all of the metastatic melanoma patients after the first PHP-M, either by SD or by PR as classified by RECIST 1.1. There was no case of progressive disease following the PHP-M treatment by chemosaturation in our cohort.

A factor found to be beneficial for a PR (in comparison to SD) was lower age (odds ratio (95% confidence interval) of age = 50 years versus age = 65 years: 0.179 (0.031, 1.040)); however, there was only weak evidence that female gender or a lower tumor volume was associated with a lower risk of SD instead of a PR.

The PHP-M procedure itself can be considered as standardized and safe, as no AEs of grades 4-5 occurred during the procedure or in the hospitalization period; thereafter, the observed adverse events up to grade IIIB were within the range of previously published data [19].

We did monitor hematological changes with a most pronounced drop in platelets; these changes were within the range of previous reports and occurred after the PHP-M procedure, frequently improving after 5–7 days. These known effects were most likely due to side effects of dilution and the use of the extracorporeal filter system, as previously described [14, 21]. A study of de Leede et al. reports an overall filter efficiency of 86% (range 71.1–95.5%) and bone marrow depression up to 80.0% of procedures [24].

However, as other authors have recommended, we suggest that patients with a tumor volume higher than 50% of the total liver volume and with abnormal liver functions (ECOG) and especially with inadequate hematological and renal functions (our exclusion limit was hemoglobin > 8 g/dL; platelets > 150 thsd/*μ*L, bilirubin ≤ 3 × upper limit) should be excluded from PHP-M [19, 23].

A wide range of other transarterial therapies are presently available for the treatment of hepatic malignancy, including bland arterial embolization, chemoembolization using a variety of chemotherapeutic agents (e.g. cisplatin) [25] or radioembolization using yttrium-90-labeled microspheres (Y90). One study to date is available reporting a direct comparison between PHP-M and SIRT (=Y90 radioembolization). This study also showed a doubled overall survival time using PHP-M chemosaturation compared with SIRT and TACE (median OS from time of treatment for PHP at 608 days versus Y90 (295 d) and CE (265 d)) [26]. However, as this was a retrospective evaluation in a single center, more work is necessary to confirm these encouraging data. For future directions, a phase III trial comparing IHP with the best alternative care in uveal melanoma is underway. The combination of 90Y-labeled microspheres with sorafenib is being studied in a phase I trial (NCT01893099), and the combination with ipilimumab is being assessed in a phase 0 study (NCT01730157).

Limitations of the reported observations comprise mainly its single-center database, its retrospective nature, and the cutoff in follow-up. Uveal melanoma is a relatively rare disease overall, and larger patient numbers are likely to be achieved only by the use of pooled data analysis from multiple centers. This retrospective approach in turn may be limited due to the inconsistency of treatment regimens and postprocedural patient care. As a result, a single center was used to minimize these confounding effects at the cost of a more limited sample size.

In conclusion, PHP-M for nonresectable liver metastasis of uveal melanoma provides a safe and locally efficient liver-directed procedure that offers patients a chance for long-term survival. PHP offers as second-line option the chance of long-term OS outcome, especially for patients with a low hepatic tumor burden.

## Figures and Tables

**Table 1:** Patients baseline data (*n* = 19).

Patient gender		
Male	11	
Female	8	
Patient age	Mean	SD
	58 years	12 years
RECIST 1.1 after first PHP		
Complete remission (CR)	0	
Partial remission (PR)	10	
Stable disease (SD)	9	
Progressive disease (PD)	0	
	Mean	SD
Time from first (positive) imaging liver to first PHP (days)	324	432
Time between first and second PHP (days)	119	145

**Table 2:** Complications after PHP chemosaturation during hospital stay according to the Clavien–Dindo classification [18].

Clavien–Dindo classification after 1 PHP	Clavien–Dindo classification after 2 PHP	Clavien–Dindo classification after 3 PHP	Clavien–Dindo classification after 4 PHP
0	0		
0	0	0	
0	0	0	
0			
IIIa	0		
0	0	0	IIIB
0	0	0	0
0	0	0	
0	0	0	
0	0		
1			
1	1		
0			
0	0		
0	0	II	
1	1		
IIIa	0	1	
0			
0			

## Data Availability

The authors make data available on request through the authors themselves. In this case, please contact the coauthor Michel Tiede, MD, at mi.tiede@asklepios.com.
